# Acute Cardiac Tamponade Following Enfortumab Vedotin Initiation in Metastatic Urothelial Carcinoma: Successful Rescue by Pericardial Fenestration

**DOI:** 10.1002/iju5.70221

**Published:** 2026-06-30

**Authors:** Hiroto Akamatsu, Yu Ishizuya, Kentaro Takezawa, Taigo Kato, Koji Hatano, Yoichi Kakuta, Atsunari Kawashima, Norio Nonomura

**Affiliations:** ^1^ Department of Urology The University of Osaka Graduate School of Medicine Suita Osaka Japan

**Keywords:** cardiac tamponade, enfortumab vedotin, pericardial effusion, pericardial fenestration, urothelial carcinoma

## Abstract

**Introduction:**

Enfortumab vedotin is a Nectin‐4‐directed antibody–drug conjugate approved for metastatic urothelial carcinoma. Cardiac adverse events such as pericardial effusion or tamponade associated with enfortumab vedotin are extremely rare.

**Case Presentation:**

A 50‐year‐old man with metastatic UC developed chest pain and dyspnea 3 days after the first EV infusion, followed by rapid progression to cardiac tamponade on Day 9. Emergency pericardiocentesis and thoracoscopic pericardial fenestration drained 2400 mL of pericardial and pleural fluid. Pericardial cytology revealed inflammatory cells without malignancy, while pleural nodules confirmed urothelial carcinoma. Infectious causes were excluded, and an acute EV‐related inflammatory reaction was suspected. EV was safely resumed on postoperative Day 20, achieving a sustained partial response for 8 months without recurrence of pericardial effusion.

**Conclusion:**

This rare case highlights the potential for acute, enfortumab vedotin‐related inflammatory pericardial effusion and emphasizes the importance of early recognition and prompt surgical intervention.

## Keynote Message

- Enfortumab vedotin is an effective treatment for advanced urothelial carcinoma but may rarely cause sudden cardiac complications.
- This case shows that early warning symptoms should not be overlooked, as rapid progression to cardiac tamponade can occur.
- Timely surgical treatment can save lives and enable patients to continue cancer therapy safely.

## 1 Introduction

Enfortumab vedotin (EV) is a Nectin‐4–directed antibody–drug conjugate approved for the treatment of advanced urothelial carcinoma after platinum‐based chemotherapy and immune checkpoint inhibitors. Although pivotal trials such as EV‐301 and EV‐302 have demonstrated its clinical efficacy, cardiac adverse events have rarely been reported [1, 2]. We describe a rare case of acute cardiac tamponade occurring immediately after EV initiation, successfully managed with thoracoscopic pericardial fenestration, which allowed safe treatment continuation and a sustained therapeutic response.

## 2 Case Presentation

A 50‐year‐old man with a history of non‐muscle‐invasive bladder cancer diagnosed 14 years earlier had undergone multiple transurethral resections. At 47 years of age, he developed left ureteral carcinoma (cT2N0M0) and underwent laparoscopic radical nephroureterectomy. One year later, robot‐assisted radical cystectomy with ileal conduit reconstruction was performed for muscle‐invasive bladder cancer (cT2N0M0). One year after cystectomy, pulmonary hilar lymph node and pulmonary metastases were detected. The patient received four cycles of gemcitabine plus cisplatin followed by maintenance avelumab. After radiographic disease progression (Figure 1A,B), enfortumab vedotin (EV) was initiated with an Eastern Cooperative Oncology Group performance status of 0. Retrospective review of the baseline CT (Figure 1B) revealed a trace pericardial effusion in addition to a small left pleural effusion. Baseline vital signs were stable. On Day 3 after the first EV infusion, the patient developed mild chest pain and dyspnea. Computed tomography revealed increased pericardial and left pleural effusions (Figure 1C). Although he remained hemodynamically stable, the cardiology team was consulted because of concern for progression to cardiac tamponade. Pericardiocentesis was considered technically difficult due to the absence of a safe puncture route, and elective thoracoscopic pericardial fenestration was planned. Opioid analgesics were administered for chest discomfort. During preoperative preparation, the patient's oxygen requirement gradually increased. On Day 9 after EV initiation, he developed hypotension, shock, and a narrowed pulse pressure. Follow‐up computed tomography demonstrated marked enlargement of the pericardial effusion (Figure 1D), leading to a diagnosis of cardiac tamponade. The patient was immediately transferred to the operating room for emergency pericardial fenestration. On arrival, he was unable to tolerate the supine position because of severe dyspnea. Endotracheal intubation and positive‐pressure ventilation were considered likely to exacerbate tamponade physiology and precipitate cardiac arrest. Therefore, urgent pericardiocentesis was performed before induction of general anesthesia, draining hemorrhagic pericardial fluid and stabilizing the circulation. Thoracoscopic pericardial fenestration and left pleural drainage were subsequently performed. Disseminated pleural nodules were observed intraoperatively, and biopsy specimens were obtained (Figure 2A). The pericardium was incised and fenestrated (Figure 2B,C), and a total of 2400 mL of pericardial and pleural fluid was drained. Cytological examination of the pericardial fluid revealed numerous inflammatory cells, including lymphocytes and histiocytes, without evidence of malignancy, and bacterial and viral cultures were negative. Histopathological analysis of the pericardial tissue demonstrated dense inflammatory cell infiltration and granulation tissue. Although degenerated atypical cells were present, no viable tumor cells were identified. Biopsy of the pleural nodules confirmed dissemination of urothelial carcinoma, with tumor cells positive for Cytokeratin AE1/AE3 and GATA3 (Figure 3A–C). After surgery, the patient was admitted to the intensive care unit. The left pleural drain, which communicated with the pericardial cavity, initially yielded approximately 700 mL/day of serosanguinous fluid. Drainage gradually decreased, and the drain tube was removed on postoperative Day 5. The patient was discharged from the intensive care unit on postoperative Day 6. Enfortumab vedotin was resumed on postoperative Day 20 at the standard dose without prophylactic systemic steroids. The patient was observed as an inpatient during the first rechallenge infusion with daily clinical assessment, serial inflammatory markers, and prompt access to echocardiography and thoracic surgical support. Follow‐up computed tomography performed 4 months after treatment resumption demonstrated sustained shrinkage of pulmonary metastases and reduction of hilar lymph node metastasis, consistent with a partial response (Figure 1E). No reaccumulation of pericardial effusion was observed, and only minimal left pleural effusion remained (Figure 1F). At 8 months after EV reinitiation, the patient continues treatment with maintained disease control.

**FIGURE 1:**
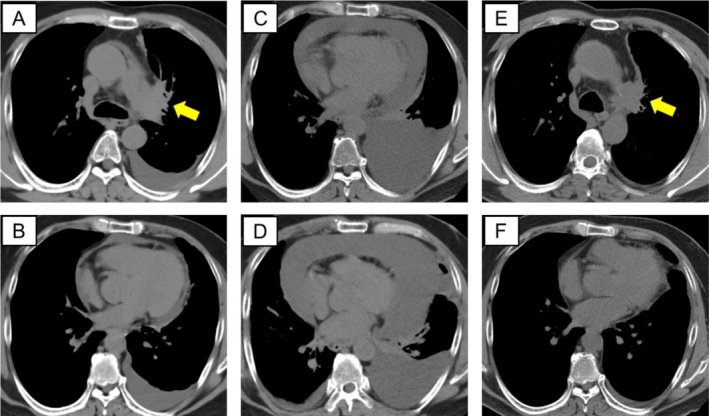
CT images during treatment. (A) Hilar lymph node (arrow) before enfortumab vedotin (EV) administration. (B) A small amount of pleural effusion and trace pericardial effusion were observed before EV administration. (C) Pericardial and pleural effusions increased 3 days after administration of EV. (D) Pericardial effusion increased significantly 8 days after EV administration. (E) Hilar lymph nodes had shrunk 4 months after starting EV. (F) Four months after starting EV, no pericardial effusion was observed, and only a small amount of pleural effusion was observed.

**FIGURE 2:**
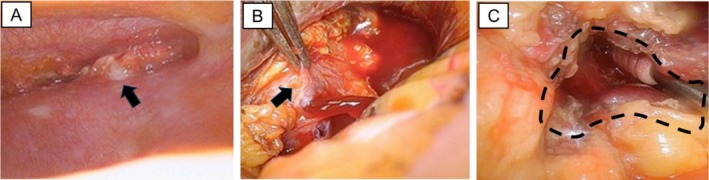
Intraoperative findings of thoracoscopic pericardial fenestration. (A) Disseminated nodules were observed in the parietal pleura (arrow). (B) Before pericardial fenestration. The arrow indicates the pericardium. (C) After pericardial fenestration. The dashed line indicates the pericardial opening.

**FIGURE 3:**
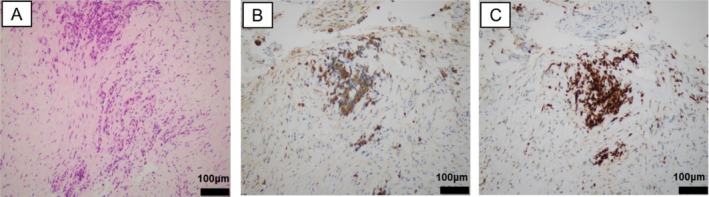
Histopathological findings of pleural disseminated nodules. (A) Hematoxylin–Eosin Staining. (B) Cytokeratin (AE1/AE3) immunohistochemical staining. (C) GATA3 immunohistochemical staining.

## 3 Discussion

Cardiac tamponade secondary to urothelial carcinoma (UC) is exceedingly rare. Only seven cases have been reported, including the present case (Table 1) [3, 4, 5, 6, 7, 8, 9]. Most occurred during metastatic disease, and prognosis has generally been poor. Our case is notable for the rapid onset of tamponade immediately after enfortumab vedotin (EV) initiation.

**TABLE 1:** Cases of cardiac tamponade due to urothelial carcinoma.

No.	Author	Year	Age/sex	Interval from UC diagnosis to pericardial dissemination	Treatment	Outcome	Follow‐up after onset of cardiac tamponade
1	Gibbs et al.	1985	70/M	36 months	Pericardial fenestration	Alive	10 days
2	Fabozzi et al.	1995	66/M	19 months	Pericardiocentesis	Cancer death	180 days
3	Spiliotopoulos et al.	2008	66/M	60 months	Pericardiocentesis	Alive	360 days
4	Khan et al.	2016	71/M	Unknown	Pericardial fenestration	Cancer death	Several days
5	Yeaman et al.	2017	70/M	Unknown	Pericardial fenestration	Cancer death	6 days
6	Palam et al.	2018	62/M	10 months	Pericardial fenestration	Alive	Unknown
7	Present case	2025	50/M	41 months	Pericardial fenestration	Alive	240 days

Although an EV‐associated inflammatory reaction is a plausible trigger for the rapid pericardial fluid accumulation, alternative explanations must be considered. Retrospective review of the baseline CT confirmed a trace pericardial effusion, and malignant pericardial involvement was clinically suspected. Pericardial cytology is imperfectly sensitive, and occult tumor infiltration cannot be excluded despite negative cytology and the absence of viable tumor cells on histology. Pericardial involvement reportedly occurs in 10%–15% of patients with pleural dissemination, likely via retrograde lymphatic spread [10, 11], and pleural dissemination was histologically confirmed in our case.

However, several findings argue against simple malignant progression as the sole cause: the temporal association with EV initiation, the rapidity of fluid accumulation, and the subsequent radiographic tumor shrinkage after EV resumption. We therefore hypothesize that EV triggered an acute inflammatory amplification—similar to a tumor lysis–associated response—on a background of pre‐existing pericardial involvement, resulting in rapid capillary leak and hemorrhagic effusion. This is supported by the predominance of inflammatory cells in the pericardial fluid, negative infectious studies, and concurrent elevations in inflammatory markers. Tumor lysis and cytokine‐mediated vascular permeability have been described as rare mechanisms of acute effusions associated with antibody–drug conjugates [12].

Regarding rechallenge, EV was resumed after multidisciplinary discussion because (1) pericardial analyses showed predominantly inflammatory changes without viable tumor cells, (2) infectious causes were excluded, and (3) the patient had limited systemic options and had progressed before EV. EV was restarted at the standard dose without prophylactic systemic steroids, with inpatient monitoring and early access to echocardiography and thoracic surgical support. This approach enabled safe continuation of effective therapy without recurrence of pericardial effusion.

This case highlights the need for early recognition of pericardial effusion during initial EV cycles, particularly in patients with thoracic metastases. Echocardiography should be promptly performed in patients presenting with dyspnea or hypotension after EV initiation. When long‐term disease control is expected, thoracoscopic pericardial fenestration may be preferable to pericardiocentesis alone, as it reduces recurrence and allows continuation of systemic therapy.

## 4 Conclusion

We report a rare case of cardiac tamponade occurring immediately after enfortumab vedotin initiation in a patient with metastatic urothelial carcinoma. The absence of malignancy or infection and the presence of inflammatory infiltration suggest a drug‐related inflammatory pericardial reaction. Prompt surgical intervention enabled recovery and continuation of effective therapy. Clinicians should be aware of this potentially life‐threatening but reversible complication.

## Ethics Statement

The authors have nothing to report.

## Consent

Informed consent was obtained from the patient for the publication of this article.

## Conflicts of Interest

The authors declare no conflicts of interest. Dr. Kentaro Takezawa is an Editorial Board member of International Journal of Urology and a co‐author of this article. To minimize bias, he was excluded from all editorial decision‐making related to the acceptance of this article for publication.

## Data Availability

The data that support the findings of this study are available on request from the corresponding author. The data are not publicly available due to privacy or ethical restrictions.
